# Four-Point Support Frame Positioning for Obese Patients Undergoing Minimally Invasive Esophagectomy. A Preliminary Institutional Experience

**DOI:** 10.5761/atcs.nm.26-00091

**Published:** 2026-07-31

**Authors:** Yusuke Asai, Naoya Okada, Takumi Yamabuki, Minoru Takada, Kentaro Kato, Yoshiyasu Ambo, Yoshihiro Kinoshita

**Affiliations:** 1 Department of Surgery, Teine Keijinkai Hospital, Sapporo, Hokkaido, Japan; 2 Esophageal Disease Center, Teine Keijinkai Hospital, Sapporo, Hokkaido, Japan

**Keywords:** patient positioning, robot-assisted surgery, prone esophagectomy

## Abstract

Prone minimally invasive esophagectomy offers several technical advantages; however, in obese patients, increased abdominal pressure may contribute to diaphragmatic elevation, leading to difficulty in first-port placement and compromised operability in the lower mediastinum. These technical challenges can be particularly relevant in robot-assisted procedures, where adequate working space and sufficient inter-arm distance are required. We describe a positioning modification using a 4-point support frame in obese patients undergoing prone minimally invasive esophagectomy. By supporting the thorax and pelvis while allowing the abdomen to hang freely, this strategy is intended to reduce abdominal compression and prevent diaphragmatic elevation. In our preliminary institutional experience, this approach facilitated port placement in the standard configuration and provided stable lower mediastinal exposure. Although based on a limited series, this positioning technique may represent a practical and reproducible option for selected obese patients undergoing prone minimally invasive esophagectomy.

## Introduction

Minimally invasive esophagectomy in the prone position (MIE-PP), first reported by Palanivelu et al. in 2006,^1)^ is a popular approach performed at different institutions. Although MIE-PP has many merits, in obese patients, the diaphragm may become elevated as a result of increased abdominal pressure, and there is a risk of diaphragmatic injury during first-port placement in the optical-view technique, as well as difficulty in manipulation of the lower mediastinum. At our institution, we observed that abdominal compression in the prone position occasionally resulted in technical difficulty during port placement and lower mediastinal dissection in obese patients. The use of a 4-point support frame was therefore introduced as a positioning modification aimed at reducing abdominal compression, allowing safe placement of ports in the standard configuration, and providing an adequate operative view even during lower mediastinal manipulation. Here, we present a practical setup for using a 4-point support frame in minimally invasive esophagectomy (MIE) for obese patients and report our preliminary institutional experience with this positioning strategy.

## Case Report

At our institution, patients undergoing prone MIE with a body mass index (BMI) of 28 kg/m^2^ or higher are considered candidates for the positioning method described below.

**Fig. 1:**
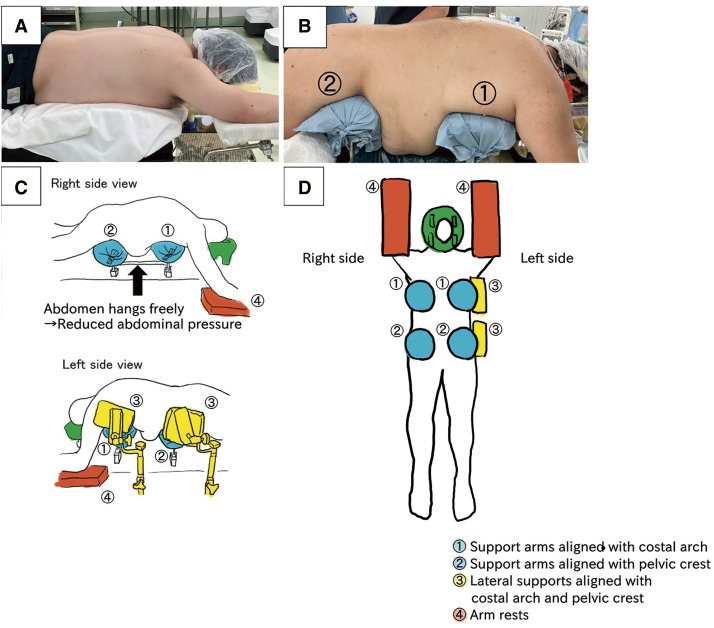
Example of patient positioning using (**A**) a conventional beanbag and (**B**–**D**) a 4-point support frame during MIE-PP. The support arms were aligned with the costal arch and pelvic crest (blue), with left-sided lateral supports to permit intraoperative rotation (yellow). Both upper limbs were fixed at 90° (orange). MIE-PP, minimally invasive esophagectomy in the prone position

In 4 consecutive patients, under general anesthesia, the patients were placed on an operating table in the prone position. Patients are usually positioned on a beanbag (**Fig. 1A**), but in this method the patients were placed on a 4-point support frame (MIZUHO, Tokyo, Japan). The arms of the frame, covered with gel pads and cloth to prevent pressure ulcers, were aligned with the costal arch and the iliac crest. To avoid skin injury caused by friction between the skin and the frame’s arm, care was taken to lift the body sufficiently when positioning it over the arms. Two lateral supports were placed on the left side to allow for rotation during surgery. For pressure relief, cushions were placed between the lateral supports and the patient’s body (**Figs. 1B**–**1D**).

A 12-mm port was inserted at the level of the sixth intercostal space along the inferior scapular line using the optical-view technique, and artificial pneumothorax was then created. No diaphragmatic injury occurred during first-port placement. The remaining ports were inserted in the usual manner under thoracoscopic visualization. Two cases were performed using a thoracoscopic approach, and the remaining 2 cases were performed under robot-assisted surgery. We were able to perform lower mediastinal manipulation with an adequate operative field. The right-sided arm of the 4-point support frame did not interfere with the surgical procedure. The anteroposterior space appeared wider, and compression by the arms did not pose any problems during the surgical procedure.

The 4-point support frame was removed during repositioning after completion of the thoracic approach. The remainder of the surgical procedure proceeded without positioning-related problems. The mean procedure time was 577 ± 47 min. No complications related to the frame, such as skin injury or rib fracture, were observed in this small series. All patients were discharged without postoperative complications.

## Discussion

**Fig. 2:**
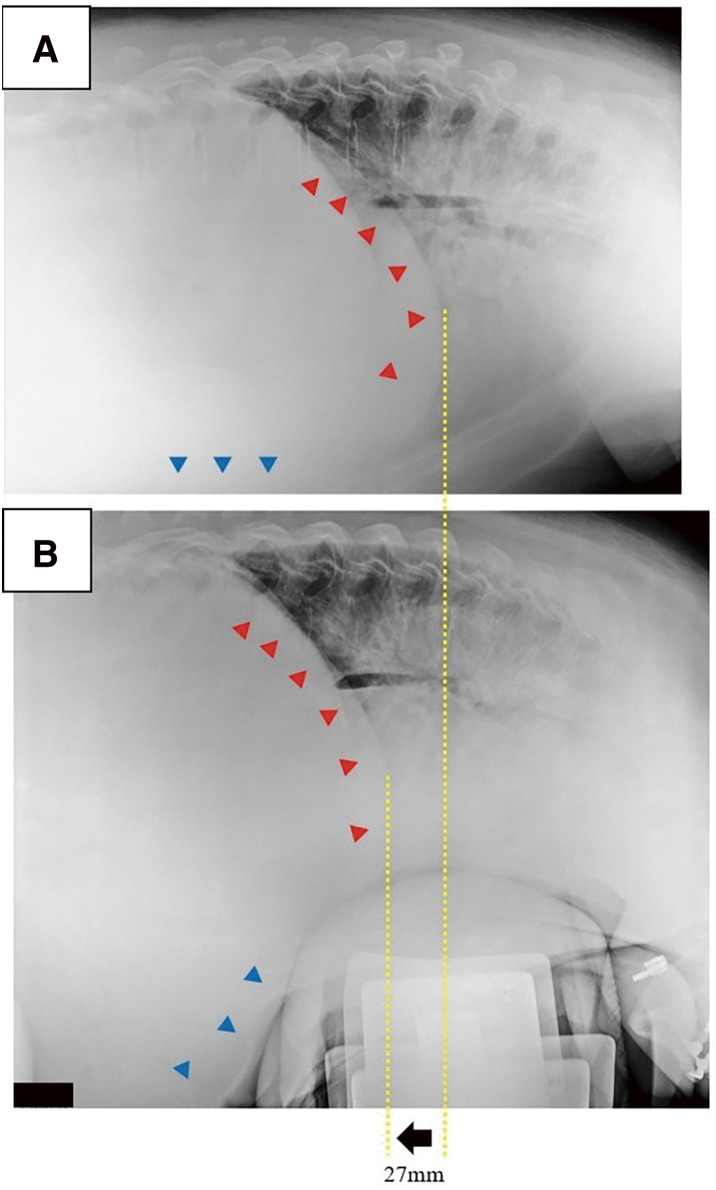
Lateral chest radiographs obtained at end-expiration in a patient with a BMI of 37 kg/m^2^ (**A**, **B**) showed a 27-mm caudal displacement of the diaphragm with the 4-point support frame compared with a mattress (red arrowheads, yellow dotted line), without change in the anteroposterior thoracic diameter, indicating reduced abdominal compression by allowing the abdomen to hang freely (blue arrowheads). BMI, body mass index

Koyanagi et al. described that the advantages of MIE-PP were as follows: a shortened learning curve; excellent surgical space; an experienced assistant is not necessarily needed; ergonomic positioning of surgical hands; theoretically improved arterial oxygenation; and one-lung ventilation is not necessarily required.^2)^ On the other hand, in obese patients, the diaphragm may be elevated as a result of increased abdominal pressure. When we performed robot-assisted esophagectomy in an obese patient in the prone position using a mattress, we encountered difficulty with first-port placement due to elevation of the diaphragm, and it took 40 minutes to complete port placement. Even after establishing artificial pneumothorax, compression by the diaphragm made surgical manipulation in the lower mediastinum difficult. We considered abdominal compression by the mattress to be a contributing factor to the diaphragmatic elevation. Although prone positioning has been widely discussed, positioning strategies specifically aimed at mitigating diaphragmatic elevation in obese patients have been limited. Therefore, the 4-point support frame was introduced to avoid increased abdominal pressure in the prone position in these cases. Compared with conventional positioning, the 4-point support frame allowed the abdomen to hang freely and appeared to reduce diaphragmatic elevation. In our institutional experience, the median time required for first-port placement was reduced from 12 minutes with conventional positioning to 7 minutes with the 4-point support frame, suggesting a potential improvement in procedural efficiency. Lower mediastinal manipulation could be performed with an adequate operative field, and no interference between the robotic arms and the frame was encountered in this small series. It has been reported in the spinal surgery field that 4-point support using the Jackson table safely reduces intra-abdominal pressure by allowing the abdomen to hang freely.^3,4)^ Our 4-point support frame is based on the same biomechanical principle and is presumed to reduce abdominal compression and subsequent diaphragmatic elevation. Unlike the Jackson table, our frame is detachable from a standard operating table and can be easily removed during patient repositioning after the thoracic procedure, making it practical for routine clinical use. In an illustrative radiographic comparison obtained in a patient with a BMI of 37 kg/m^2^, caudal displacement of the diaphragmatic dome was observed when the frame was used (**Fig. 2**). Both radiographs were obtained with the participant in the same position during voluntary breath-holding at end-expiration. Although this assessment was not systematic, it is consistent with the proposed mechanical rationale.

The use of this technique in robot-assisted minimally invasive esophagectomy (RAMIE) may offer 3 major advantages by mitigating diaphragmatic elevation. First, it may facilitate safer port placement. In our institution, the first port is routinely inserted at the sixth intercostal space along the subscapular line using the optical-view technique. Even in obese patients, this technique may allow the first port to be placed safely without modifying the standard approach. Second, the port for arm 1 can be inserted more caudally, thereby increasing the distance between robotic arms. If the sole objective is to avoid diaphragmatic injury during first-port placement, one might argue that placing the first port at a more cranial level would suffice. However, in the cranial region, the subcutaneous fat layer is thicker, increasing the risk of misplacement away from the intended intercostal space. Particularly in RAMIE, where the range of motion is markedly restricted by the ribs, maintaining the planned intercostal level may help secure adequate inter-arm distance and reduce instrument interference. Third, this technique may improve operability in the lower mediastinum. By reducing diaphragmatic elevation, a more stable operative field may be maintained, potentially facilitating dissection. Although the present series is limited in size, the intraoperative findings suggest the feasibility and potential utility of this positioning strategy.

On the other hand, there are 2 potential disadvantages of using the frame. First, it has a risk of intraoperative pressure injury because of the smaller areas of body surface contact.^5)^ Therefore, it is necessary to align the arms with the ribs and pelvis, and to cover the arms with gel pads and cloth to prevent pressure injury. Second, patient repositioning may become more complex. In emergency situations requiring conversion to thoracotomy, turning the patient to the lateral decubitus position can be challenging. Accordingly, thorough preoperative planning, clear team communication, and rehearsal of the conversion process are recommended to ensure safety.

## Conclusion

The 4-point support frame may provide a practical and reproducible positioning strategy for obese patients undergoing prone minimally invasive esophagectomy. By reducing abdominal pressure and potentially reducing diaphragmatic elevation, it may facilitate safe first-port placement and may improve lower mediastinal operability. This technique may be a useful positioning option that provides technical consistency when selectively applied to obese patients.
